# Taste perception and food preferences in patients with diabetic foot ulcers before and after hyperbaric oxygen therapy

**DOI:** 10.1038/s41387-022-00219-x

**Published:** 2022-10-05

**Authors:** Magdalena Hartman-Petrycka, Grzegorz Knefel, Agata Lebiedowska, Mariusz Nowak, Barbara Błońska-Fajfrowska

**Affiliations:** 1 Department of Basic Biomedical Science, Faculty of Pharmaceutical Sciences in Sosnowiec, Medical University of Silesia, Katowice, Poland; 2 Dr Stanisław Sakiel Centre for Burn Treatment, Siemianowice Śląskie, Poland

**Keywords:** Nutrition, Diabetes complications

## Abstract

**Objective:**

The aim of the study was to evaluate the effect of hyperbaric oxygen therapy (HBOT) on taste perception and food preferences in patients with diabetic foot ulcers.

**Methods:**

The study involved 75 healthy people (Group C) and 23 patients with diabetic foot ulcers before HBOT (Group Db) and after 25–30 HBOT treatments (Group Da) (2.5 ATA, 87 min). The sip and spit method was used to examine the taste perception for 5 basic flavours. Food preferences were studied using photographs of dishes.

**Results:**

The recognition thresholds in Group C were lower than in Group Db for 5 basic flavours. The taste intensity in Group C was higher than in Group Db for: 0.1% and 1.0% monosodium glutamate, 0.02% citric acid, and 0.002% quinine hydrochloride. The hedonic response in Group C was more negative than in Group Db for: 0.18% sodium chloride, 0.3% monosodium glutamate and 0.1% citric acid. The pleasure derived from eating in Group C was lower than in Group Db for sour and salty products. The recognition thresholds in Group Db were higher than in Group Da for umami and sour. The taste intensity in Group Db was lower than in Group Da for: 0.1%, 0.3% and 1.0% monosodium glutamate. The pleasure derived from eating in Group Db was higher than in Group Da for chocolate and crisps.

**Conclusions:**

In people with diabetic foot ulcers, an impaired all 5 basic tastes occurred with different food preferences compared to healthy people. HBOT causes beneficial changes resulting in increased sensitivity to umami and sour taste as well as a decrease in the pleasure derived from eating chocolate and crisps.

## Introduction

Type 2 diabetes is a diet-dependent disease with improper nutrition and a sedentary lifestyle being important contributory factors to its development [1]. The chemosensory function of taste has a big impact on eating behaviour. As a result of information provided by the mouth, decisions about the final selection of food are made. This information helps to protect the body from consuming harmful substances and encourages the consumption of substances which are rich in nutrients [2]. People with diabetes have a number of complications that can result in the distortion of taste perception and thus affect diet and compound abnormal eating behaviour [3].

Adverse changes occur within the mouth, which is often overlooked when treating patients with diabetes [4]. Meanwhile, Candida-associated denture stomatitis, burning mouth syndrome, dryness of the oral mucosa, angular cheilitis and glossitis are much more common in patients with type 2 diabetes than in healthy people, and the frequency of these disorders depends on glycaemic control [4]. All pathologies within the mucous membrane of the tongue and soft palate as well as salivation along with diabetic neuropathy may be the cause of the dysfunction in the perception of taste sensations in all the basic flavours. A direct relationship has been demonstrated between chemosensory dysfunction and the severity of vascular complications [5], peripheral neuropathy and microalbuminuria [6]. People with diabetes can develop diabetic foot complications due to peripheral arterial disease and peripheral neuropathy. One of the methods used to treat this complication is hyperbaric oxygen therapy (HBOT) where patients breathe 100% oxygen at increased air pressure, >1 atmosphere absolute (1ATA) [7].

The treatment of taste perception disorders is a challenge. Positive results in this area have been obtained by the oral administration of zinc [8, 9], intranasal theophylline treatment [10] and magnetic stimulation of the trigeminal nerve [11]. The best results, however, are obtained by treating the underlying disease entity that has led to the taste disorder [12].

Given the wide range of effects that HBOT has on the body and its positive effects in the treatment of diabetic foot complications [7, 13, 14], the question that arises is—are there also changes in taste perception and food preferences? Authors of earlier studies have indicated the beneficial effect of HBOT in this area in a non-homogeneous group of patients with non-healing wounds [15]. This study focuses on diabetic patients with diabetic foot ulcers. Due to the strong relationship between the level of complications and a patient’s diet, the assessment of the impact of hyperbaric therapy on taste sensitivity and food preferences in this group is justified.

## Methods

### Key elements of the study design

The study involved healthy people (Group C) and patients with diabetic foot ulcers before HBOT (Group Db) and after 25–30 HBOT treatments (Group Da). An examination of perception of taste sensations of the five basic flavours and a study of food preferences were carried out. The taste recognition threshold, taste intensity and hedonic perception of taste sensations were determined. The taste perception and food preferences were compared in Group C and Group Db as well as in Group Db and Group Da.

### The study participants

**Fig. 1:**
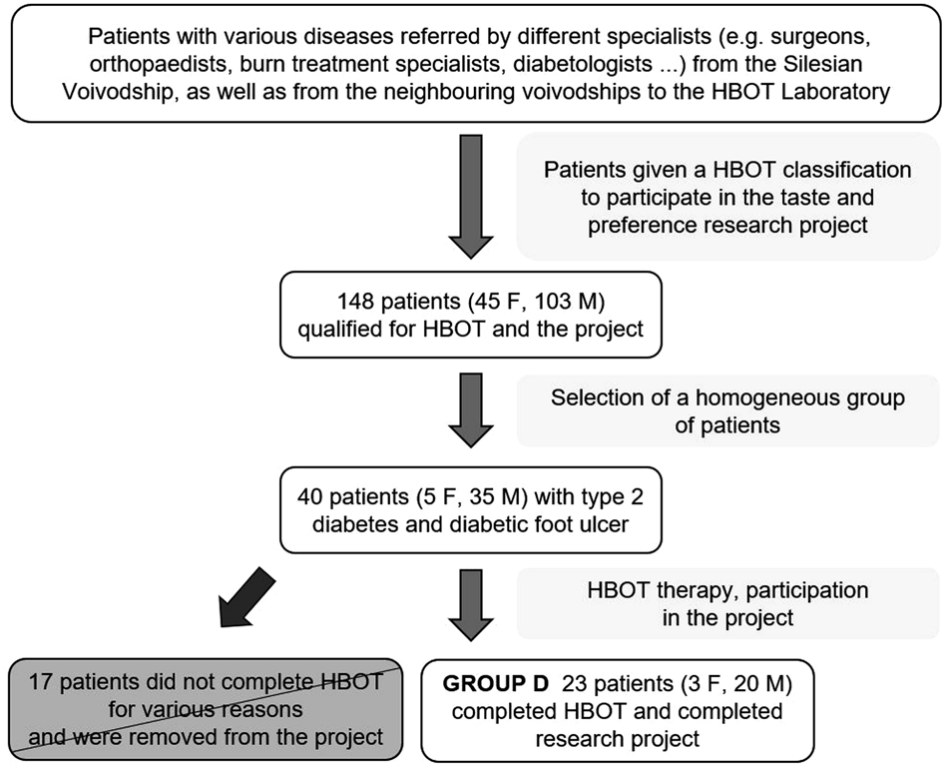
Flowchart of the study patients recruitment. Patient enrolment for group D.

The results analysed in this study were part of a project in which the influence of hyperbaric oxygen therapy on the perception of taste sensations and food preferences was assessed. Patient enrolment in the study is shown in Fig. 1.

Patients were referred to the hyperbaric oxygen laboratory by various specialists, most often surgeons, orthopaedists, burn treatment specialists and diabetologists from the Silesian Voivodeship and its neighbouring voivodeships. In the Hyperbaric Oxygen Laboratory, a hyperbaric physician made the decision to refer a patient for hyperbaric therapy after studying the patient’s medical history and their current state of health. The inclusion criteria included the presence of a condition with indications for HBOT treatment and eligibility for the National Health Fund reimbursement list. The exclusion criteria included: health conditions that preclude using hyperbaric oxygen therapy, health conditions requiring immediate hyperbaric therapy i.e., carbon monoxide poisoning, location of wounds or a disability that makes it difficult to take taste samples orally, any injuries to the oral mucosa, hearing loss or any disease that impedes communication with the patient, the patient’s inability to understand the taste examination procedure or refusal to consent to participate in the research. As part of the entire project, initial tests of taste and food preferences were carried out among 148 people in various states of health. Most of them suffered from non-healing wounds of different etiologies, but some people had been referred for hyperbaric therapy due to other issues, e.g., bone necrosis. From this group, a homogeneous group of 40 patients with diabetes mellitus type 2 and diabetic foot ulcers was selected.

17 people stopped the HBOT for various reasons, e.g., due to a general feeling of malaise during the treatment, colds, occurrence of additional health issues and problems with access to the hyperbaric facility. Finally, 23 people with type 2 diabetes and diabetic foot ulcers—3 women and 20 men who formed the D group (Fig. 1) and 75 healthy people—11 women and 64 men who formed the Control Group C participated in the full study of taste and food preferences. Group C was matched to Group D in terms of age and BMI (Table 1). The health status of the patients with both diabetic mellitus and diabetic foot ulcers was based on the patient’s interview as presented in Table 2. A specialist in hyperbaric medicine assessed the condition of the wound before starting HBOT, on completion of HBOT and once a week during HBOT. Before and after HBOT, the Ulcer Stage was determined according to The University of Texas Diabetic Foot Ulcer Classification System (Table 3). Planimetric photos of the ulcer before and after HBOT were also taken, which became the basis for measuring the ulcer area (Table 3). In five patients (21.7%) the changes related to the impact of HBOT were assessed only visually, without measuring the ulcer area because of the location of the ulcer. In 20 people, 87.0%, the area of the ulcer decreased after HBOT, in two people (8.7%) the area of the ulcer increased and in one person, the area of the ulcer did not visibly change.

**Table 1:** Characteristics of Control Group (C) and patients with diabetic mellitus with diabetic foot ulcers before (Db) and after (Da) hyperbaric oxygen therapy.

Group	Characteristics	Mean	sd	Min	Q1	Me	Q3	Max
C (*N* = 75)	Age (years)	57.2	6.5	45.0	53.0	57.0	61.0	73.0
	BMI	28.0	3.4	21.8	25.8	27.7	30.2	41.4
	Hunger level*	2.7	0.7	1.0	2.0	3.0	3.0	4.0
Db (*N* = 23)	Age (years)	57.4	7.6	46.0	49.0	59.0	63.0	72.0
	BMI	29.6	4.7	22.7	25.0	28.7	33.1	37.6
	Time since diagnosis of diabetes (years)	12.8	9.6	0.3	5.0	10.0	20.0	36.0
	Time since appearance of the ulcer (months)	11.0	10.3	1.0	4.0	5.5	18.0	33.0
	Blood glucose level 1st test day (mg/dl)	168.9	49.4	70.0	135.0	167.0	208.0	248.0
	Blood glucose level 2nd test day (mg/dl)	159.1	63.1	57.0	104.0	158.0	185.0	295.0
	Hunger level*	3.4	0.5	3.0	3.0	3.0	4.0	4.0
Da (*N* = 23)	Blood glucose level 1st test day (mg/dl)	159.9	45.6	105.0	126.0	149.5	182.0	267.0
	Blood glucose level 2nd test day (mg/dl)	152.9	63.4	77.0	103.0	137.5	210.0	272.0
	Hunger level*	3.3	0.6	2.0	3.0	3.0	4.0	4.0

*N* number of study participants, *sd* standard deviation, *Q1* first quartile, *Me* median, *Q3* third quartile, *Min* minimum, *Max* maximum.

Hunger level* (1—very hungry, 2—hungry, 3—medium state, 4—full, 5—very full).

**Table 2:** Treatment, diabetes’ complications and comorbidities of patients with diabetic mellitus and diabetic foot ulcers (Group D).

Treatment	*N* (%)	Diabetes’ complications and comorbidities	*N* (%)	Ulcer	*N* (%)
Insulin and hypoglycaemia oral agents/GLP1 agonist injection	21 (91,3)	Neuropathy	3 (13,0)	Previous ulcer in another location	6 (26,1)
Hypoglycemic oral agents/ GLP1 agonist injection only	1 (4,3)	Chronic kidney disease	1 (4,3)	Previous amputation in another location	4 (17,4)
Only by diet	1 (4,3)	Retinopathy	2 (8,7)	Previous HBO due to the ulcer in another location	4 (17,4)
		Peripheral artery disease	18 (78,3)	Previous amputation in current location of the ulcer	5 (21,7)
		Varicose veins/venous thrombosis	8 (34,8)		
		Other additional (from 1 to 4 comorbidities, average 2.1 disease)	11 (47,8)		

*N* (%)—number and percentage.

**Table 3:** Ulcer characteristics in patients with diabetic mellitus and diabetic foot ulcers (Group D).

The current location of the ulcer	*N* (%)	Ulcer stage according to The University of Texas Diabetic Foot Ulcer Classification System			Ulcer area [mm^2^]		
		Class	Before HBOT *N* (%)	After HBOT *N* (%)		Before HBOT	After HBOT
Lower leg	1 (4.3)	0A	0 (0)	1 (4.3)	Median	4.183	1.139
Foot	15 (65.2)	1A	10 (43.5)	10 (43.5)	Mean	9.331	3.137
Foot and toes	5 (21.7)	1B	2 (8.7)	1 (4.3)	St. dev.	20.808	5.251
Toes	2 (8.7)	2A	5 (21.7)	7 (30.4)	Minimum	0.103	0.00
		2B	3 (13.0)	2 (8.7)	Maximum	92.773	22.302
		2D	1 (4.3)	0 (0)			
		3A	0 (0)	1 (4.3)			
		3B	1 (4.3)	1 (4.3)			
		3D	1 (4.3)	0 (0)			

*N* (%)—number and percentage.

### Study procedure

The research project was approved by the Bioethics Committee of the Medical University of Silesia. The study was conducted in accordance with the Helsinki Declaration, and every participant provided written consent after being informed of the aim, protocol, and methodology of the study.

Group C participated in the gustatory test once, on two consecutive days in a gustometric laboratory at the Department of Basic Biomedical Sciences, Medical, University of Silesia, Katowice, Poland. On the first day, the perception of three basic taste categories was examined, and on the second day, the two remaining tastes and food preference was tested. The study was performed in the morning on an empty stomach.

Group D participated in the gustatory tests in two steps, before the hyperbaric therapy (Group Db) and at the end of the therapy (Group Da), after having undergone at least 25 sessions of treatment in the hyperbaric chamber; for example before the 26th or 27th session. The hyperbaric treatment took place in the oxygen hyperbaric facility in the ‘Dr. Stanislaw Sakiel Centre for Burn Treatment’ in Siemianowice Śląskie. Gustatory tests were carried out in the morning. Diabetic patients were allowed to eat breakfast 2 hours before the gustatory test, because there was a concern that hypoglycaemia could occur when travelling to the hospital and during the test. All diabetic patients had their glucose levels measured prior to every chamber entry, as shown in Table 1.

The HBOT included 25–30 sessions which took place on consecutive days excluding weekends and holidays. A multiplace hyperbaric chamber was used for the treatment. Each session was conducted at 2.5 ATA (1 atmosphere absolute - ATA) pressure using compressed air and lasted for 87 min. Patients breathed 100% oxygen through a fitted mask covering the nose and mouth. Exhaled air was discharged through valves connected to the chamber’s pneumatic system.

### Gustatory tests

In the gustatory tests, we studied taste recognition threshold, taste intensity, and hedonic perception of the basic taste categories. These parameters were measured according to standard ISO 3972 procedures [16].

For taste recognition threshold, taste intensity, and hedonic perception tests aqueous solutions of five substances were used: sucrose solutions for sweet, sodium chloride for salty, monosodium glutamate for umami, citric acid for sour and quinine hydrochloride for bitter (Table 4). The samples of 15 ml solutions were administered in transparent cups and labelled with randomised three-digit codes. The participants knew neither the type of substance used nor the coding system. The patients tasted the samples according to the sip and spit method, in the order shown in Table 4. The patients evaluated their taste sensations in response to each sample from a series of 10 concentrations and marked one of the answers: no sensation, sweet, salty, bitter, sour, umami. The taste recognition threshold was the lowest concentration of substance correctly recognised by the patient.

**Table 4:** Concentrations of taste solutions used for the gustometric investigations.

	Concentrations					Type of examination
Order of solutions	Sodium chloride [g/l]	Sucrose [g/l]	Monosodium glutamate [g/l]	Citric acid [g/l]	Quinine hydrochloride [µg/l]	
1	0.16	0.34	0.08	0.0036	0.0222	Taste recognition threshold
2	0.24	0.55	0.12	0.0057	0.0378	
3	0.34	0.94	0.17	0.0088	0.0642	
4	0.48	1.56	0.24	0.0138	0.1092	
5	0.69	2.59	0.34	0.0216	0.1856	
6	0.98	4.32	0.49	0.0338	0.3156	
7	1.40	7.20	0.70	0.0528	0.5365	
8	2.00	12.00	1.00	0.0830	0.9121	
9	2.85	20.00	1.43	0.1300	1.5505	
10	4.07	33.33	2.04	0.2000	2.6359	

Order of solutions	Sodium chloride [%]	Sucrose [%]	Monosodium glutamate [%]	Citric acid [%]	Quinine hydrochloride [%]	
I	0.18	1	0.1	0.02	0.001	Taste intensity and hedonic response
II	0.36	10	0.3	0.04	0.002	
III	0.90	30	1.0	0.10	0.005	

After a 15-min break, the patients tasted a series of three suprathreshold concentrations (Table 4). They recorded the intensity of their taste perceptions on a 10-cm linear analogue scale with the starting point of ‘0’ for no flavour and the end point ’10’ for maximum intensity of flavour. They also marked the degree of pleasure derived from a taste sensation on a linear analogue scale with extreme points described as the most unpleasant (−5.0), the most pleasant (5.0) and the middle point referred to as neutral (0). The results were obtained by measuring the distance from the zero point on the scale to a subject’s mark.

### Food preference tests

Participants were shown 20 pages of colour photographs showing certain dishes: vegetables and salads, fruits, sweet desserts, chocolate, milk dishes, cheese, dumplings, pasta, bread, beef and pork, poultry, broth, egg dishes, fish dishes, seafood, fast food, salty products, crisps, sour products, spicy dishes. After being shown the photos, they were asked to determine how pleasant the dish appeared to them. The answers were marked on 10-cm linear analogue scale with the starting point marked “0” (unpleasant) and the end point “10” (extremely pleasant).

### Data analysis

Data was saved and analysed using Microsoft Excel 2010 and Statistica 12.0 software. The Mann–Whitney U test was used to compare the results of the Control (Group C) with the diabetic patients before hyperbaric therapy (Group Db). The effect of HBOT on the taste recognition threshold, the intensity, hedonic perception and food preferences in diabetic patients (Group Db vs Group Da) were assessed using the Wilcoxon signed-rank test. The statistically significant difference was set at *p* < 0.05.

## Results

The taste sensitivity in people with type II diabetes and diabetic foot ulcers before hyperbaric therapy (Group Db) (*N* = 23) was lower compared to the healthy people (Group C) (*N* = 75) for all 5 basic flavours (salty *p* < 0.05, sweet *p* < 0.01, umami *p* < 0.01, sour *p* < 0.001, bitter *p* < 0.01) (Fig. 1).

The median value in Group C and Group Db group for the recognition threshold for salty was 6 and 8 (0.68 g/l and 2.00 g/l sodium chloride), sweet 7 and 8 (7.20 g/l and 12.00 g/l sucrose, respectively), umami 8 and 11 (1.00 g/l monosodium glutamate and no sensation), acid 5 and 9 (0.0216 g/l and 0.1300 g/l citric acid) and bitter 6 and 9 (0.3156 µg/l and 1.5505 µg/l quinine hydrochloride).

**Fig. 2:**
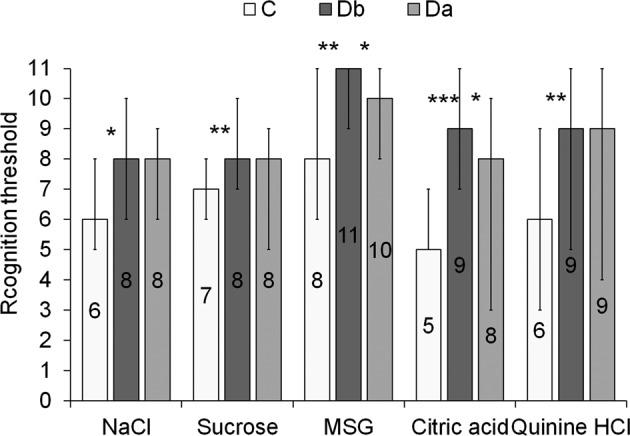
Recognition threshold (the sample with lowest concentration resulting in the correct recognition of a taste category) for salty (NaCl), sweet (sucrose), umami (monosodium glutamate or MSG), sour (citric acid), and bitter (quinine hydrochloride or HCl) solutions in Control Group (C), in people with diabetic mellitus before (Db) and after (Da) hyperbaric oxygen therapy. Number 11—no sensation; box—median, whisker—interquartile range; *p < 0.05, **p < 0.01, **p < 0.001.

In patients with diabetes taste sensitivity increased slightly for the salty, sweet and bitter taste and increased significantly for umami (*p* < 0.05) and sour (*p* < 0.05) (Fig. 2) as a result of hyperbaric therapy. The median value in people with diabetes before (Group Db) and after (Group Da) hyperbaric therapy, for umami taste recognition threshold was 11 and 10 (no sensation and 2.04 g/l monosodium glutamate) and for sour taste 9 and 8 (0.1300 g/l and 0.0830 g/l citric acid).

In healthy people, the intensity of umami taste sensation for 0.1% monosodium glutamate solutions was significantly higher than in people with diabetes (*p* < 0.05) (Table 5). The median value for the intensity of taste sensation for the solution of this concentration was 1.9 in Group C and 1.0 in Group Db. Similarly, in Group C, the intensity of taste sensation for 1.0% monosodium glutamate solution was higher than in Db group (*p* < 0.05), the median value of intensity of this taste was 7.2 in Group C and 5.9 in Group Db, respectively. Healthy people could taste the sourness of a 0.02% citric acid solution with greater intensity than people with diabetes before HBOT (*p* < 0.05). The median value for the intensity of taste sensation for this taste in Group C was 4.3 and in Group Db 2.4. Also, the bitter taste from 0.002% quinine hydrochloride solution was perceived more intensively in healthy people than in diabetic patients before hyperbaric treatment (*p* < 0.05). The median value for the intensity of taste sensation for this taste in Group C was 7.0 and in Group Db 5.1.

**Table 5:** Intensity of taste sensations for specific concentration of salty (NaCl—sodium chloride), sweet (sucrose), umami (MSG—monosodium glutamate), sour (citric acid), and bitter (Quinine HCl—quinine hydrochloride) solutions in Control Group (C) and patients with diabetes mellitus before (Db) and after (Da) hyperbaric treatment.

	C						Db						Da				
	Min	Q1	Me	Q3	Max	p_CvsDb_	Min	Q1	Me	Q3	Max	p_DbvsDa_	Min	Q1	Me	Q3	Max
NaCl																	
0.18%	0.0	0.7	1.4	3.1	6.3	ns	0.0	0.3	0.9	3.1	5.0	ns	0.0	0.0	1.0	2.0	2.9
0.36%	0.0	3.3	4.8	6.0	10.0	ns	0.7	2.1	4.0	5.2	8.0	ns	0.0	3.2	4.7	6.0	8.0
0.90%	1.3	6.9	8.5	9.8	10.0	ns	5.0	7.2	8.0	9.4	10.0	ns	1.7	7.1	8.6	9.4	10.0
Sucrose																	
1%	0.0	0.9	1.8	3.2	6.7	ns	0.0	1.0	1.5	3.8	6.4	ns	0.0	0.4	1.2	2.7	6.8
10%	3.5	6.2	7.3	8.7	10.0	ns	4.1	6.8	7.7	9.5	10.0	ns	3.9	5.9	7.7	9.0	10.0
30%	6.0	9.1	9.8	10.0	10.0	ns	7.8	9.7	10.0	10.0	10.0	ns	0.0	9.0	10.0	10.0	10.0
MSG																	
0.1%	0.0	0.7	1.9	3.7	8.3	<0.05	0.0	0.0	1.0	2.2	5.0	<0.05	0.0	0.8	2.0	3.2	6.4
0.3%	0.2	2.2	4.8	6.4	9.5	=0.069	0.0	1.1	3.4	5.3	7.0	<0.05	0.0	2.3	4.0	6.4	8.2
1.0%	0.5	5.1	7.2	9.0	10.0	<0.05	0.0	2.5	5.9	7.3	10.0	<0.05	0.4	5.3	7.0	8.7	10.0
Citric acid																	
0.02%	0.7	2.6	4.3	5.2	7.9	<0.05	0.0	1.5	2.4	5.0	8.3	ns	0.9	2.1	3.1	4.0	8.0
0.04%	0.4	5.1	6.6	7.7	9.8	ns	0.9	3.2	6.1	7.1	9.5	ns	1.7	4.9	5.2	6.5	9.1
0.10%	1.8	7.7	8.7	9.6	10.0	ns	3.8	6.4	8.0	9.6	10.0	ns	3.2	7.4	8.2	9.3	10.0
Quinine HCl																	
0.001%	0.5	2.2	4.2	6.0	9.1	ns	0.0	1.5	3.0	5.0	10.0	ns	0.0	2.0	3.3	5.2	9.1
0.002%	1.7	6.0	7.0	8.4	10.0	<0.05	0.7	4.4	5.1	7.6	10.0	ns	1.0	5.2	6.3	8.0	9.4
0.005%	4.9	8.5	9.3	10.0	10.0	ns	5.0	7.5	9.0	10.0	10.0	ns	3.4	8.1	9.6	10.0	10.0

*Q1* first quartile, *Me* median, *Q3* third quartile, *Min* minimum, *Max* maximum.

HBOT affected the intensity of the umami taste sensation for all three threshold levels of monosodium glutamate: 0.1% (*p* < 0.05), 0.3% (*p* < 0.05) and 1.0% (*p* < 0.05). The median value for the intensity of the umami taste sensation of these solutions in people with diabetes before hyperbaric therapy (Group Db) was: 1.0; 3.4 and 5.9, and after therapy (Group Da) 2.0; 4.0 and 7.0.

There are differences for the hedonic response to 0.18% sodium chloride (*p* < 0.05), 0.3% sodium glutamate (*p* < 0.05) and 0.10% citric acid between healthy people (Group C) and those with diabetes before hyperbaric therapy (Group Db) (Table 6). The minimum value (Min) and first quartile value (Q1) for the hedonic response to a 0.18% NaCl solution in Group C were −4.5 and −0.1, and in Group Db: 0.0 and 0.0. The minimum value and first quartile value for the hedonic response to a 0.3% MSG solution in Group C were −5.0 and −1.2, and in Group Db: −4.3 and 0.0. The first quartile and median for the hedonic evaluation of the 0.10% citric acid solution in Group C were −4.4 and −2.4, and in Group Db: −2.8 and 0.0.

**Table 6:** Hedonic response for specific concentration of salty (NaCl—sodium chloride), sweet (sucrose), umami (MSG—monosodium glutamate), sour (citric acid), and bitter (Quinine HCl—quinine hydrochloride) solutions in Control Group (C) and patients with diabetes mellitus before (Db) and after (Da) hyperbaric treatment.

	C						Db						Da				
	Min	Q1	Me	Q3	Max	p_CvsDb_	Min	Q1	Me	Q3	Max	p_DbvsDa_	Min	Q1	Me	Q3	Max
NaCl																	
0.18%	−4.5	−0.1	0.0	0.0	5.0	<0.05	0.0	0.0	0.0	0.0	2.8	ns	−2.0	0.0	0.0	0.5	4.6
0.36%	−4.9	−1.4	0.0	0.1	5.0	ns	−4.0	0.0	0.0	0.0	1.5	=0.055	−2.8	0.0	0.0	0.8	4.4
0.90%	−5.0	−3.7	−1.5	0.5	5.0	ns	−5.0	−3.6	0.0	1.5	4.2	ns	−5.0	−4.0	−1.0	0.0	2.1
Sucrose																	
1%	−3.3	0.0	0.0	1.1	5.0	ns	−2.6	0.0	0.0	1.3	3.1	ns	0.0	0.0	0.0	1.2	3.4
10%	−4.4	0.0	1.3	2.6	5.0	ns	−4.2	0.8	2.5	3.6	5.0	ns	−1.4	0.0	2.1	2.9	4.8
30%	−5.0	−3.0	0.8	3.8	5.0	ns	−5.0	−4.6	1.7	4.2	5.0	ns	−5.0	−2.4	1.2	3.5	5.0
MSG																	
0.1%	−5.0	−0.1	0.0	0.0	5.0	ns	−3.6	0.0	0.0	0.1	3.0	ns	−1.1	0.0	0.0	0.2	2.9
0.3%	−5.0	−1.2	0.0	0.5	5.0	<0.05	−4.3	0.0	0.0	1.6	2.7	ns	−3.4	0.0	0.0	1.0	3.7
1.0%	−5.0	−3.4	−0.6	0.8	5.0	ns	−5.0	−0.1	0.0	1.1	4.0	ns	−4.6	−1.2	0.0	1.0	3.7
Citric acid																	
0.02%	−5.0	−0.5	0.0	0.8	3.8	ns	−3.0	−0.6	0.0	0.0	3.3	ns	−5.0	−0.4	0.0	0.0	3.5
0.04%	−5.0	−1.6	−0.7	0.1	3.4	ns	−3.8	−1.9	0.0	0.7	4.7	ns	−5.0	−1.4	0.0	0.0	4.1
0.10%	−5.0	−4.4	−2.4	0.0	5.0	<0.05	−5.0	−2.8	0.0	0.0	4.7	ns	−5.0	−4.0	−1.3	0.0	2.2
Quinine HCl																	
0.001%	−5.0	−2.1	−0.9	0.0	0.4	ns	−5.0	−1.6	0.0	0.0	0.6	ns	−5.0	−2.2	−0.5	0.0	0.0
0.002%	−5.0	−3.7	−2.4	−1.2	0.0	ns	−5.0	−3.0	−1.5	0.0	3.7	ns	−4.6	−3.5	−2.3	−1.5	0.0
0.005%	−5.0	−4.9	−4.4	−3.1	0.8	ns	−5.0	−5.0	−4.0	−1.8	4.1	ns	−5.0	−5.0	−4.4	−3.3	0.0

*Q1* first quartile, *Me* median, *Q3* third quartile, *Min* minimum, *Max* maximum.

HBOT did not have a statistically significant effect on the hedonic response of the tested flavours in the suprathreshold concentrations.

The food preferences in healthy people and people with diabetes are different. The median value for the pleasure derived from eating sour products in Group C was 5.9 and in Group Db − 7.3 (*p* < 0.05), while the median value for the pleasure derived from eating salty products in Group C was 2.8 and in Group Db − 5.0 (*p* < 0.05) (Table 7).

**Table 7:** Food preference in control group (C) and patients with diabetes mellitus before (Db) and after (Da) hyperbaric treatment.

	C						Db						Da				
	Min	Q1	Me	Q3	Max	p_CvsDb_	Min	Q1	Me	Q3	Max	p_DbvsDa_	Min	Q1	Me	Q3	Max
Broth	0.4	7.5	9.1	10.0	10.0	ns	0.1	7.3	8.6	10.0	10.0	ns	2.5	5.6	7.5	10.0	10.0
Vegetables and salads	0.3	6.1	8.0	9.7	10.0	ns	1.0	5.8	8.5	9.9	10.0	ns	0.6	5.1	8.3	9.0	10.0
Fruits	0.0	7.0	8.9	10.0	10.0	ns	1.0	5.3	8.5	10.0	10.0	ns	0.8	5.9	7.8	9.0	10.0
Fish dishes	0.0	5.2	7.8	9.8	10.0	ns	0.4	5.7	8.0	9.8	10.0	ns	4.0	7.3	8.8	10.0	10.0
Cheese	0.0	5.3	7.2	8.3	10.0	ns	0.1	4.7	8.0	8.7	10.0	ns	0.6	5.3	6.4	7.5	10.0
Egg dishes	0.4	5.4	7.0	9.1	10.0	ns	0.0	6.0	7.9	8.5	10.0	ns	4.0	5.1	6.9	8.0	10.0
Poultry	0.0	6.5	7.8	8.9	10.0	ns	0.4	6.8	7.8	10.0	10.0	ns	0.7	7.0	7.9	9.5	10.0
Bread	3.6	6.7	8.3	9.5	10.0	ns	2.5	5.5	7.6	9.6	10.0	ns	1.0	5.8	7.5	8.2	10.0
Beef and pork	2.3	6.7	7.9	9.4	10.0	ns	0.0	7.0	7.5	8.8	10.0	ns	1.7	7.2	8.1	9.0	10.0
Sour products	0.0	2.5	5.9	8.0	10.0	<0.05	0.8	6.3	7.3	9.7	10.0	ns	1.9	6.5	7.8	8.1	10.0
Milk dishes	0.4	4.6	5.9	7.8	10.0	ns	0.6	5.6	7.2	8.9	10.0	ns	0.8	5.1	7.3	7.9	10.0
Spicy dishes	0.0	1.2	4.2	7.2	10.0	ns	0.0	4.3	7.0	8.6	10.0	ns	0.7	3.0	6.3	7.5	10.0
Chocolate	0.5	5.1	7.8	9.6	10.0	ns	0.0	3.2	6.7	8.1	10.0	<0.05	0.0	2.4	5.1	6.5	10.0
Dumplings	0.4	3.9	5.7	9.1	10.0	ns	0.5	4.5	6.7	9.8	10.0	ns	1.5	5.0	8.0	8.4	10.0
Sweet desserts	0.5	6.7	8.7	9.9	10.0	ns	0.0	5.0	6.2	10.0	10.0	ns	1.1	4.6	5.5	8.0	10.0
Salty products	0.0	0.9	2.8	4.8	10.0	<0.05	0.0	2.8	5.0	5.5	10.0	ns	0.0	2.0	3.7	5.5	7.8
Pasta	0.3	3.2	5.2	7.5	10.0	ns	1.1	3.0	4.9	7.9	10.0	ns	1.8	3.2	6.2	8.0	10.0
Crisps	0	0.9	1.9	4.7	10	ns	0.0	0.4	1.3	5.3	7.5	=0.050	0.0	0.0	1.1	2.8	5.7
Seafood	0.0	0.0	0.7	5.2	10.0	ns	0.0	0.0	1.0	3.2	10.0	ns	0.0	0.5	2.3	6.9	9.7
Fast food	0.0	0.0	1.2	3.4	10.0	ns	0.0	0.0	0.7	2.8	6.9	ns	0.0	0.4	1.5	2.6	9.3

*Q1* first quartile, *Me* median, *Q3* third quartile, *Min* minimum, *Max* maximum.

Under the influence of hyperbaric therapy in patients with diabetes, the pleasure derived from eating chocolate decreased (*p* < 0.05). Before hyperbaric therapy in people with diabetes (Group Db), the median value and third quartile value of pleasure from eating chocolate were 6.7 and 8.1 and after treatment (Group Da) 5.1 and 6.5. The pleasure derived from eating crisps (*p* = 0.050) also tended to diminish. Before the treatment (Group Db), the median and third quartile for the pleasure of eating crisps were 1.3 and 5.3 and after the treatment (Group Da) 1.1 and 2.8.

## Discussion

In this study, prior to hyperbaric therapy, a number of negative differences in the perception of the five basic tastes were demonstrated by type II diabetes with diabetic foot ulcers when compared to the controls. These concerned the recognition threshold for basic tastes and the intensity of taste sensation of suprathreshold concentrations. Differences were also noted in the hedonic response of the suprathreshold concentrations of some basic tastes as well as in food preferences. The use of hyperbaric therapy increased chemosensory sensitivity to some extent, and diminished the pleasure derived from eating chocolate and crisps.

An increase in the recognition threshold for saltiness in people with diabetic foot ulcers, and differences for the hedonic response to 0.18% NaCl were found. In diabetics, the lack of reaction to this taste was predominant and no one indicated that the taste of this solution was unpleasant, while for more than 25% of healthy people, the salty taste of 0.18% NaCl was assessed negatively. These results may explain why people with diabetic foot ulcers derive greater pleasure from eating salty snacks such as salty sticks (e.g., salty breadsticks, pretzels) or crackers. This can translate into greater/more frequent consumption of this type of snack and other salty products. Excessive salt intake is harmful, and its effects depend on individual sensitivity to sodium chloride [17]. According to WHO recommendations [18], adults are advised to consume <2 g of sodium per day (5 g salt per day) to reduce hypertension and the risk of cardiovascular disease, stroke and coronary heart disease. The altered sense of saltiness in people with diabetic foot ulcers observed in this paper, corresponds with the results of Gondvikar et al. [19]. These studies included patients with type 2 diabetes with controlled and uncontrolled glycaemia. In both groups, a lower recognition threshold for the salty taste was detected.

In this study, people with type 2 diabetes showed an increase in the recognition threshold for sweet taste, but this did not translate into differences in the hedonic response to the suprathreshold concentrations of sucrose. According to Yu et al. [20], the recognition threshold for sweet taste in people with type 2 diabetes was higher than in healthy people, which is consistent with the results contained in this study. In addition, Yu et al. observed that people with type 2 diabetes preferred lower concentrations of sucrose than healthy people. Moreover, healthy people had a clear negative correlation between the recognition threshold and preferences for sweet solutions, which was not found in people with type 2 diabetes. The research methodology of Yu et al. differs from the methodology presented in this paper, so differences may arise regarding sweet taste preferences in people with diabetes in these two studies. Studies by Gondvikar et al. [19] in patients with type 2 diabetes confirm the occurrence of taste disturbances. Wasalathanthri et al. [21] also tested sweet taste sensitivity in pre-diabetics and diabetics. The sweet taste recognition threshold in pre-diabetic and diabetic people did not differ significantly from healthy people, but increasing the recognition threshold for sweet taste and decreasing the intensity of suprathreshold ratings indicate a sweet taste dysfunction. The effectiveness of chemosensory function of sweet taste is extremely important. In people with glucose intolerance/diabetes, a sweet taste dysfunction increased the incidence of vascular complications and other complications such as ischaemic heart disease, diabetic nephropathy and diabetic retinopathy [5].

In this study, patients with diabetic foot ulcers have an increased recognition threshold for umami taste. In addition, the assessment of three suprathreshold concentrations of monosodium glutamate showed a lower intensity of their taste sensation, and the hedonic response to a 0.3% solution of monosodium glutamate was more positive. No results confirming or challenging the results of this study were found in available literature.

Some light can be shed on this subject by results, in knockout mouse models. TRPM5−/− mice show a reduction in glucose-induced insulin secretion and a significantly reduced response to umami taste as well as sweet taste and bitter taste. Perhaps in patients with type 2 diabetes, umami, sweet and bitter taste dysfunctions are associated with decreased TRPM5 expression [22].

In people with diabetic foot ulcers, the recognition threshold for sour taste increases. In addition, they perceived 0.02% citric acid solution as less intense and 0.10% as less unpleasant. All these differences may contribute to the fact that people with diabetes like sour products more than healthy people. Sensitivity to sour taste in people with type 2 diabetes was studied by Gondvikar et al. [19]. They showed that the recognition threshold for sour taste in people with diabetes was higher than in the control.

In this study, an increase in the recognition threshold for bitter taste and a decrease in the intensity of sensation for 0.002% quinine hydrochloride was demonstrated in patients with diabetic foot ulcers. In the publication of Gondvikar et al. [19] it was not shown that the recognition threshold for bitter taste in diabetic people differed from healthy people. However, the dysfunction of bitter taste perception at the front and back of the tongue and on the soft palate was revealed by a different test method—the spatial taste test.

Furthermore, research using electrogustometry has shown reduced taste sensitivity in patients with type 2 diabetes in correlation with the duration of the disease [6].

Aside from diabetic foot ulcers treatment, the HBOT partly fulfilled the authors’ expectations by improving the sense of taste and changing food preferences. Although the effects of hyperbaric therapy made no difference to the perception of salty, sweet and bitter taste, there was an improvement in umami and sour taste sensitivity and beneficial changes in food preferences.

After HBOT in patients with diabetic foot ulcers, the recognition threshold for umami taste decreased and the intensity of taste sensation for suprathreshold concentrations (0.1% and 1.0%) of monosodium glutamate increased. Changes in perception of umami taste correlate with the lower pleasure derived from eating crisps. These unhealthy snacks are usually spiced with monosodium glutamate and/or disodium 5-ribonucleotide which give food its umami flavour. Increasing the umami taste sensitivity could make the taste of crisps too intense and reduce the pleasure derived from eating them. However, given the many factors that might affect food preferences and the numerous hyperbaric therapy effects on the human body, this is only one of many possible hypotheses which could account for changes in the pleasure derived from eating crisps. The diabetic patients post HBOT declared less pleasure from eating chocolate products, but there was no change in sweet taste sensitivity. Nevertheless, the recognition threshold for the sour taste increased while the pleasure from eating sour products did not change. This confirms the multifactorial causes of changes in food preferences.

In people with diabetic foot ulcers, HBOT produces a number of positive effects on the body including a decrease in HbA1c levels and leucocyte counts [23]. In addition, studies on diabetic rats showed a decrease in blood glucose, and triglyceride levels as a result of HBOT [24, 25]. Studies in adult insulin-dependent diabetes mellitus patients [26] found a reduction of total cholesterol, triglycerides and low-density lipoprotein. In addition, there was an increase in expression of insulin-like growth factor binding protein 1 and a decrease in insulin level. HBOT causes beneficial metabolic changes, such as an increase in the oxidative capacity of the skeletal muscle and a slowing down of the age-related decrease in oxidative capacity of the skeletal muscle, known as a hypothetical mechanism of counteracting insulin resistance [24]. Increased serum levels of IL-10, IL-6, IFN-γ, IL-4 and adiponectin have been shown in patients with diabetic foot ulcers treated with hyperbaric oxygen [27]. In healthy rats during HBOT an increase in the expression of leptin and visfatin genes, as well as IL-1β and IL-10 were also demonstrated [28]. Taste sensitivity and food preferences are dependent on hormonal activity and metabolic changes in the body [2], so, perhaps the changes resulting from HBOT, described above, are the basis for improving taste sensitivity and reducing the pleasure of eating unhealthy snacks in patients with diabetic foot ulcers.

Due to the lack of publications (to the best of the authors’ knowledge) on the effect of HBOT on taste sensitivity, oral mucosa and salivation in patients with type 2 diabetes, this issue was analysed based on the results of tests on irradiated people and animals. Gerlach et al. [29] have shown that in patients receiving radiation therapy for head and neck cancer, the use of HBOT reduced swallowing difficulties, decreased mouth dryness, improved taste sensitivity and increased saliva volume. Studies about the effect of HBOT on irradiated oral mucosa showed that 6 months after treatment microvessel density and the cross-sectional area of blood vessels increased in the sub-epithelial area and deeper connective tissue [30]. Spiegelberg et al. [31] confirmed the positive effect of HBOT on damaged tissues in irradiated mice. Despite the fact that the oral mucosa in patients after radiation therapy and in patients with diabetic foot ulcers certainly shows a different degree and type of dysfunction, perhaps the mechanisms supporting its regeneration after HBOT, described in people after radiation therapy, partially explain the improvement in the perception of taste sensations in diabetics. The assumption that HBOT improves the condition of the oral mucosa in people with diabetes also indirectly confirms the effects obtained in the diabetic foot ulcers itself in which fibrosis and angiogenesis can occur [27]. In addition, studies using diabetic mice have shown not only angiogenesis but also an increase in stem cells proliferation [32].

The methodology of taste recognition testing in the world of science is varied. There is still no so-called gold standard. Each method has its advantages and disadvantages. For example, a difference between objective methods (recording of evoked potentials in encephalography (EEG) and magnetoencephalography (MEG) or modern fMRI imaging (functional magnetic resonance imaging)) and subjective methods, such as specific gustometry and electrogustometry exists [33]. Objective methods do not require the patient to answer questions, however, they do require complex devices and there may be difficulties in analysing the results. Subjective methods do not require complicated devices, however, they require the patient to understand the procedure and to cooperate. In the research of specific gustometry, different concentrations of solutions and different flavours are used, as well as different techniques for the application of flavours. Depending on the procedure used, the results may be inconsistent. In this study, the method described in the ISO 3972 procedures [16] was used. This was modified, based on previous research experience, by increasing the number of samples to also include people with lower taste sensitivity. In order to eliminate methodological differences and obtain reliable results, the same procedures were applied during the taste examination both in the control group and in the diabetic patients with a diabetic foot ulcer. The disturbances in the perception of taste between the control group and the diabetic patients with a diabetic foot ulcer, as shown in this research, are consistent with the picture presented in literature [19–21]. This, in turn, confirms the reliability of the obtained results and the procedure used in the taste examination.

The clinical significance of the results obtained in this research is difficult to determine. Formation of food preferences and eating behaviour is very complicated. Food preferences depend on numerous, interdependent factors, e.g., on the characteristics of the consumed product (colour, temperature, texture, serving aesthetics), the social context of the meal, as well as the psychological and biological characteristics of a consumer [34]. The chemosensory sensitivity improvement certainly affects the nutritional behaviour of diabetic patients, but the range of this effect cannot be determined based on the presented results. In order to assess the clinical significance of the observed changes in taste sensitivity and food preferences due to hyperbaric oxygen therapy, the patients’ diet should be fully monitored at least 1 week prior to treatment and for an extended period of time after treatment. It can only be assumed that, to some extent, the improvement in the metabolic status of diabetic patients described by other researchers [23, 26] is based on the mechanisms presented in this study.

In people with diabetes, healthy eating behaviours are important in preventing the development of complications. This study shows that patients with diabetic foot ulcers have a distorted perception of taste sensations and to some extent different food preferences. HBOT increases taste sensitivity and alters the patient’s food preferences to more beneficial ones. Unfortunately, we do not know to what extent the diet of patients with diabetes will change, which is the main limitation of this study. Further research is required to fully explain the effects of HBOT on nutrition. However, based on the positive effects described in this paper and in other studies, the more frequent use of HBOT as an adjunct therapy in complications of type 2 diabetes is worth considering.

This study involved patients with advanced diabetes. This meant having a diagnosis of diabetes at least 10 years ago and where some complications, including non-healing wounds, had already appeared. At the same time, it should be remembered that those patients with severe complications, where there was a problem with understanding the test procedure, collecting taste samples or marking answers, were not invited to participate in the study. Due to the particular character of the studied group, the obtained results can only refer to a relatively narrow group of people suffering from type II diabetes. The development of diabetes mellitus type II can be slowed down with appropriate treatment at the initial stage before complications occur and patients can maintain relatively good health for many years. On the other hand, diabetes which is not treated properly is associated with numerous and serious complications that may be a direct cause of death. Observation of the influence of hyperbaric oxygen therapy on the taste sensitivity and food preferences in people at different stages of the disease could indicate the target group in which such interactions bring the best results. The limitation of the study is the lack of glycated haemoglobin tests. This parameter, used in monitoring glycaemia and the effectiveness of diabetes treatment, could complement the clinical picture of patients participating in the study and serve as a reference point in the interpretation of the results.

## Conclusions

People with severe stage type II diabetes and complications in the form of diabetic foot ulcers have an impaired sense of taste for salt, sweet, umami, sour and bitter as well as altered food preferences compared to healthy people. HBOT in people with diabetes, at the stage mentioned above, causes beneficial changes resulting in an increased sensitivity to umami and sour taste as well as a decrease in the pleasure derived from eating chocolate products and crisps. Further studies are needed to assess the clinical relevance of the results as well as to assess the impact that the severity of diabetes complications has on taste sensitivity and food preferences. In addition, research is needed on the level of impact that HBOT has on the sense of taste and food preferences in those diabetic patients suffering with varying degrees of complication severity.

## Data Availability

The datasets generated during and/or analysed during the current study are available from the corresponding author on reasonable request.

## References

[1] International Diabetes Federation. Available from https://www.idf.org/. Accessed 19 Nov 2019.

[2] Loper HB, La Sala M, Dotson C, Steinle N (2015). Taste perception, associated hormonal modulation, and nutrient intake. Nutr Rev.

[3] Gondivkar SM, Indurkar A, Degwekar S, Bhowate R (2009). Evaluation of gustatory function in patients with diabetes mellitus type 2. Oral Surg Oral Med Oral Pathol Oral Radio Endod.

[4] Dorocka-Bobkowska B, Zozulinska-Ziolkiewicz D, Wierusz-Wysocka B, Hedzelek W, Szumala-Kakol A, Budtz-Jörgensen E (2010). Candida-associated denture stomatitis in type 2 diabetes mellitus. Diabetes Res Clin Pr.

[5] Tsujimoto T, Imai K, Kanda S, Kakei M, Kajio H, Sugiyama T (2016). Sweet taste disorder and vascular complications in patients with abnormal glucose tolerance. Int J Cardiol.

[6] Le Floch JP, Le Lièvre G, Labroue M, Peynègre R, Perlemuter L (1992). Early detection of diabetic patients at risk of developing degenerative complications using electric gustometry: a five-year follow-up study. Eur J Med.

[7] Erdoğan A, Düzgün AP, Erdoğan K, Özkan MB, Coşkun F (2018). Efficacy of hyperbaric oxygen therapy in diabetic foot ulcers based on Wagner classification. J Foot Ankle Surg.

[8] Mahajan SK, Prasad AS, Lambujon J, Abbasi AA, Briggs WA, McDonald F (1980). Improvement of uremic hypogeusia by zinc: a double-blind study. Am J Clin Nutr.

[9] Yoshida S, Endo S, Tomita H (1991). A double-blind study of the therapeutic efficacy of zinc gluconate on taste disorder. Auris Nasus Larynx.

[10] Henkin RI, Schultz M, Minnick-Poppe L (2012). Intranasal theophylline treatment of 419 hyposmia and hypogeusia: a pilot study. Arch Otolaryngol Head Neck Surg.

[11] Henkin RI, Potolicchio SJ, Levy LM (2011). Improvement in smell and taste dysfunction after repetitive transcranial magnetic stimulation. Am J Otolaryngol.

[12] Henkin RI, Levy LM, Fordyce A (2013). Taste and smell function in chronic disease: a review of clinical and biochemical evaluations of taste and smell dysfunction in over 5000 patients at The Taste and Smell Clinic in Washington, DC. Am J Otolaryngol.

[13] Salama SE, Eldeeb AE, Elbarbary AH, Abdelghany SE (2019). Adjuvant hyperbaric oxygen therapy enhances healing of nonischemic diabetic foot ulcers compared with standard wound care alone. Int J Low Extrem Wounds.

[14] Pérez-Panero A, Ruiz-Muñoz M, Cuesta-Vargas A, Gónzalez-Sánchez M (2019). Prevention, assessment, diagnosis and management of diabetic foot based on clinical practice guidelines: a systematic review. Medicine.

[15] Hartman-Petrycka M, Knefel G, Lebiedowska A, Kosmala J, Klimacka-Nawrota E, Kawecki M (2016). Alterations in taste perception as a result of hyperbaric oxygen therapy. Appetite.

[16] The Polish Committee for Standardization. Sensory analysis. Methodology. Method for taste sensitivity research. Polish Norm PN ISO 3972.1998. [In Polish: Polski Komitet Normalizacyjny. Analiza sensoryczna. Metodologia. Metoda sprawdzania wrażliwości smakowej. Polska Norma PN ISO 3972.1998]. Poland; 2016.

[17] Agócs R, Sugár D, Szabó AJ. Is too much salt harmful? Yes Pediatr Nephrol. 2019. 10.1007/s00467-019-04387-4. 10.1007/s00467-019-04387-4 PMC7384997 31781959

[18] WHO. Guideline: Sodium intake for adults and children. Geneva: World Health Organization (WHO); 2012. 23658998

[19] Gondivkar SM, Indurkar A, Degwekar S, Bhowate R (2009). Evaluation of gustatory function in patients with diabetesmellitus type 2. Oral Surg Oral Med Oral Pathol Oral Radio Endod.

[20] Yu JH, Shin MS, Lee JR, Choi JH, Koh EH, Lee WJ (2014). Decreased sucrose preference in patients with type 2 diabetes mellitus. Diabetes Res Clin Pr.

[21] Wasalathanthri S, Hettiarachchi P, Prathapan S (2014). Sweet taste sensitivity in pre-diabetics, diabetics and normoglycemic controls: a comparative cross sectional study. BMC Endocr Disord.

[22] Vennekens R, Mesuere M, Philippaert K (2018). TRPM5 in the battle against diabetes and obesity. Acta Physiol.

[23] Irawan H, Semadi IN, Widiana I. A pilot study of short-duration hyperbaric oxygen therapy to improve HbA1c, leukocyte, and serum creatinine in patients with diabetic foot ulcer Wagner 3–4. Sci World J. 2018. 10.1155/2018/6425857. 10.1155/2018/6425857 PMC6109474 30158840

[24] Ishihara A (2019). Mild hyperbaric oxygen: mechanisms and effects. J Physiol Sci.

[25] Fujita N, Goto N, Nakamura T, Nino W, Ono T, Nishijo H, et al. Hyperbaric normoxia improved glucose metabolism and decreased inflammation in obese diabetic rat. J Diabetes Res. 2019;2019. 10.1155/2019/2694215. 10.1155/2019/2694215 PMC6885850 31828157

[26] Resanović I, Gluvić Z, Zarić B, Sudar-Milovanović E, Vučić V, Arsić A (2020). Effect of hyperbaric oxygen therapy on fatty acid composition and insulin-like growth factor binding protein 1 in adult insulin-dependent diabetes mellitus patients: a pilot study. Can J Diabetes.

[27] Anguiano-Hernandez YM, Contreras-Mendez L, de Los Angeles Hernandez-Cueto M, Oz-Medina JEM, Santillan-Verde MA, Barbosa-Cabrera RE (2019). Modification of HIF-1α, NF-aκB, IGFBP-3, VEGF and adiponectin in diabetic foot ulcers treated with hyperbaric oxygen. Undersea Hyperb Med.

[28] Şen H, Erbağ G, Ovalı MA, Öztopuz RÖ, Uzun M (2016). Investigation of endocrine and immunological response in fat tissue to hyperbaric oxygen administration in rats. Cell Mol Biol.

[29] Gerlach N, Barkhuysen R, Kaanders JHAM, Janssens GORJ, Sterk W, Merkx MAW (2008). The effect of hyperbaric oxygen therapy on quality of life in oral and oropharyngeal cancer patients treated with radiotherapy. Int J Oral Maxillofac Surg.

[30] Svalestad J, Hellem S, Thorsen E, Johannessen AC (2015). Effect of hyperbaric oxygen treatment on irradiated oral mucosa: microvessel density. Int J Oral Maxillofac Surg.

[31] Spiegelberg L, Braks JAM, Djasim UM, Farrell E, van der Wal KGH, Wolvius EB (2014). Effects of hyperbaric oxygen therapy on the viability of irradiated soft head and neck tissues in mice. Oral Dis.

[32] Peña-Villalobos I, Casanova-Maldonado I, Lois P, Prieto C, Pizarro C, Lattus J (2018). Hyperbaric oxygen increases stem cell proliferation, angiogenesis and wound-healing ability of WJ-MSCs in diabetic mice. Front Physiol.

[33] Klimacka-Nawrot E, Suchecka W (2008). Metody badań wrażliwości smakowej. Wiad lek.

[34] Wądołowska L, Babicz-Zielińska E, Czarnocińska J (2008). Food choice models and their relation with food preferences and eating frequency in the Polish population: POFPRES study. Food Policy.

